# Analysis of clinical and genetic features in an adolescent patient with primary ciliary dyskinesia induced by homozygous mutation in the *RSPH4A* gene: a case report

**DOI:** 10.3389/fped.2025.1630730

**Published:** 2025-08-07

**Authors:** Wanting Xu, Yan Yang, Lan Kang, Ling Guo, Jing Liu, Yan Zeng, Lei Li, Ai Chen, Rong Zhang, Wenbin Dong

**Affiliations:** ^1^ Division of Neonatology, Department of Pediatrics, The Affiliated Hospital of Southwest Medical University, Luzhou, Sichuan, China; ^2^ Department of Perinatology, The Affiliated Hospital of Southwest Medical University, Luzhou, Sichuan, China; ^3^ Pediatrics, Sichuan Clinical Research Center for Birth Defects, Luzhou, Sichuan, China; ^4^ Pediatrics, Chengdu Second People’s Hospital, Chengdu, Sichuan, China

**Keywords:** adolescent patient, primary ciliary dyskinesia, homozygous mutations, *RSPH4A*, case report

## Abstract

Primary ciliary dyskinesia (PCD) is a rare genetically heterogeneous disorder characterized by dysfunctional motile cilia, with or without detectable ultrastructural abnormalities. This study focuses on a homozygous mutation in the rare radial spoke head component 4A (*RSPH4A*) gene in a Chinese adolescent girl with PCD. The patient, an 11-year and 3-month-old girl, developed neonatal pneumonia after birth and gradually presented with persistent perennial rhinitis and recurrent productive cough. Lung CT scan indicated bronchiectasis, and whole-exome sequencing (WES) exhibited a novel pathogenic homozygous c.351dup (p. Pro118Serfs*2) frameshift mutation in the *RSPH4A* gene. A literature review reported that 21 pathogenic variants in *RSPH4A* have been discovered. WES recognized disease-causing mutations in PCD, and c.351dup (p. Pro118Serfs*2) frameshift mutation in *RSPH4A* may become a hotspot in Chinese patients.

## Introduction

Primary ciliary dyskinesia (PCD) is a rare, congenital disease induced by mutations in genes that encode ciliary components, primarily manifesting as recurrent respiratory infections, chronic sinusitis, and secretory otitis media (1). PCD is diagnosed based on a combination of genetic sequence testing, high-speed video microscopy tests for cilia beat frequency and pattern, transmission electron microscopy (TEM), and nasal nitric oxide (2). Its prevalence varies between 1/15,000 and 1/30,000 live births, with >50 genes and over 2,000 causative mutations (3). The proteins that make up the motile cilia structure in the upper and lower respiratory tracts are impacted by genetic mutations (4).

The *RSPH4A* gene (c.921+3_921+6delAAGT) was first identified as a pathogenic variant in Puerto Rico and linked to the ancestor's haplotype in 2013 (5), and there were only a few reports of variants in China. Prior research verified the presence of *RSPH4A* in the radial spoke heads of cilia's microtubules. Its variations mainly damage the ciliary ultrastructure and result in the absence of radial spoke heads, which impairs mucus clearance (6). In this study, we reported a novel homozygous frameshift mutation in *RSPH4A* (GRCh37/hg19:chr6:116938137, Exon:1/6, NM_001010892.3 c.351dup p.Pro118Serfs*2), which has never been documented before in the literature on patients with *RSPH4A*-related disease. Additionally, we conducted a literature review to clarify the clinical characteristics and provide compound variations for early identification of this PCD genotype.

## Case report

**Figure 1:**
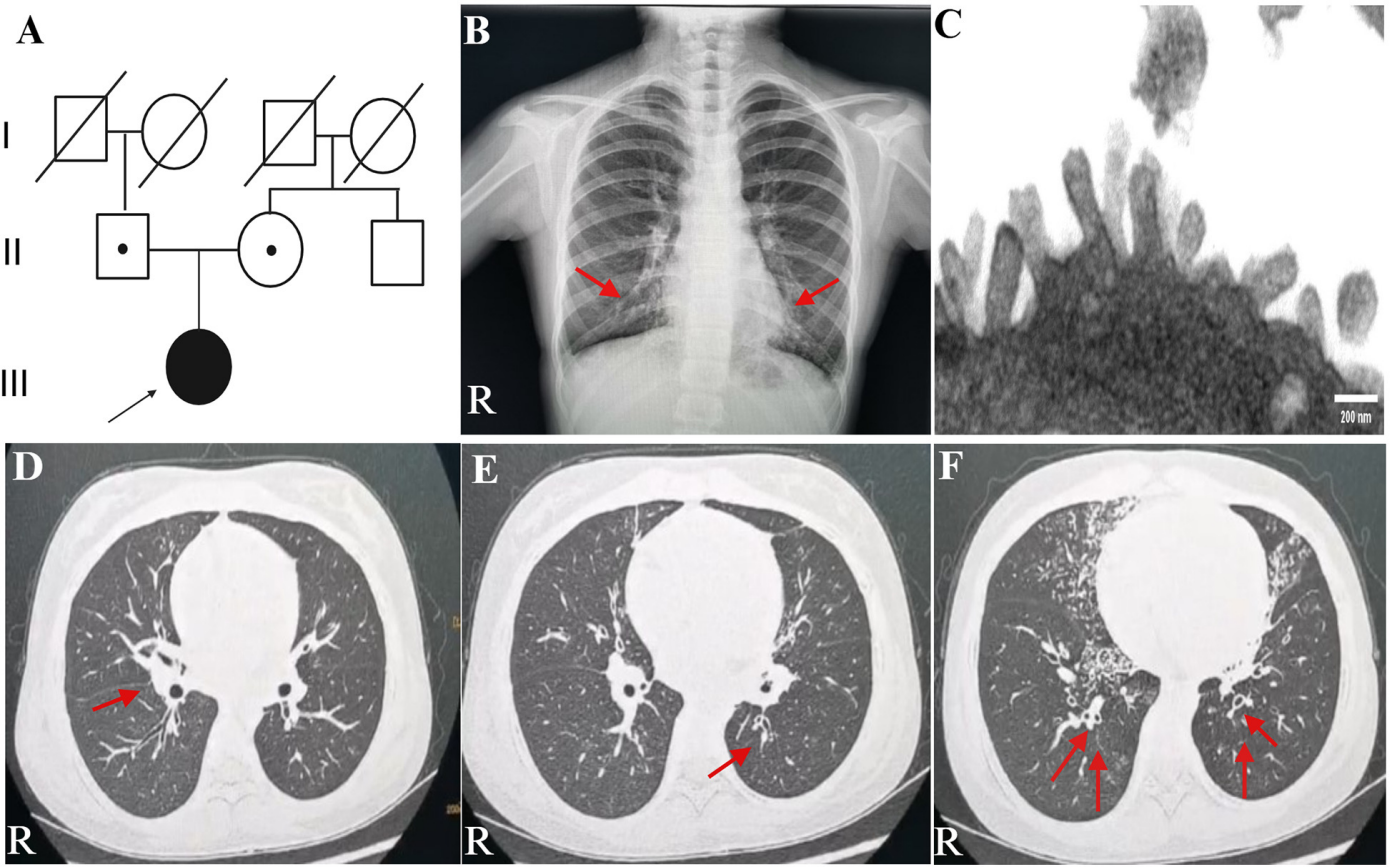
Family history and clinical characteristics of the patient. **(A)** This PCD patient came from a non-consanguineous family, and her parents showed no clinical symptoms. The teenage female proband inherited a homozygous mutation from both heterozygous carrier parents. The proband is identified by the arrow. **(B)** Chest radiograph indicated bilateral pleural thickening, inflammation of lower fields of both lungs, and no ectopic heart. Inflammation infiltration images are shown by the red arrows. **(C)** TEM indicated microvilli on the epithelial surface. **(D–F)** Axial view: lung CT scan indicated infections in the middle lobe of the right lung and the lower lingual segment of the upper lobe of the left lung and inflammation of lower lobes of both lungs, with slightly dilated bronchi in the lower lobes of both lungs. As is shown by the red arrows. PCD, primary ciliary dyskinesia; R, right; TEM, transmission electron microscope.

A teenage girl, aged 11 years and 3 months, was admitted to the respiratory department of our hospital with a 1-week history of cough and 1-day history of fever. She came from a non-consanguineous family, according to the family history, and her parents showed no symptoms (Figure 1A). Born at term, the patient was admitted to a neonatal unit and diagnosed as neonatal pneumonia at 24 h after birth. She had no history of prolonged oxygen dependency and displayed situs solitus. She had persistent perennial rhinitis and a recurrent productive cough since she was 7 years old. She takes montelukast orally every day and continuously inhales powdered budesonide and formoterol fumarate. Additionally, the patient was diagnosed with sinusitis and secretory otitis media by the otolaryngologist based on nasal obstruction, purulent nasal discharge for over 4 years, and the results of the otoscopy examination. Her motor, cognitive, and language development was normal, with no hydrocephalus. At 11 years old, she was admitted to our respiratory department for community-acquired pneumonia. Chest radiograph indicated bilateral pleural thickening and inflammation of lower fields of both lungs (Figure 1B). Bronchoscopy revealed a lot of white and viscous secretions in the bronchial cavities bilaterally. A bacterial culture of bronchoalveolar lavage fluid (BALF) exhibited *Pseudomonas aeruginosa*. Transmission electron microscope (TEM) showed microvilli with few cilia on the epithelial surface (Figure 1C). Electrocardiogram, echocardiography, myocardial enzymes, liver and kidney function tests, plasma electrolytes, serum mycoplasma and chlamydia antibodies, and immunoglobulin levels were unremarkable.

On physical examination, the adolescent patient indicated no shortness of breath. Auscultation exhibited coarse to moderate dampness and phlegm rales.

### Auxiliary examination

**Figure 2:**
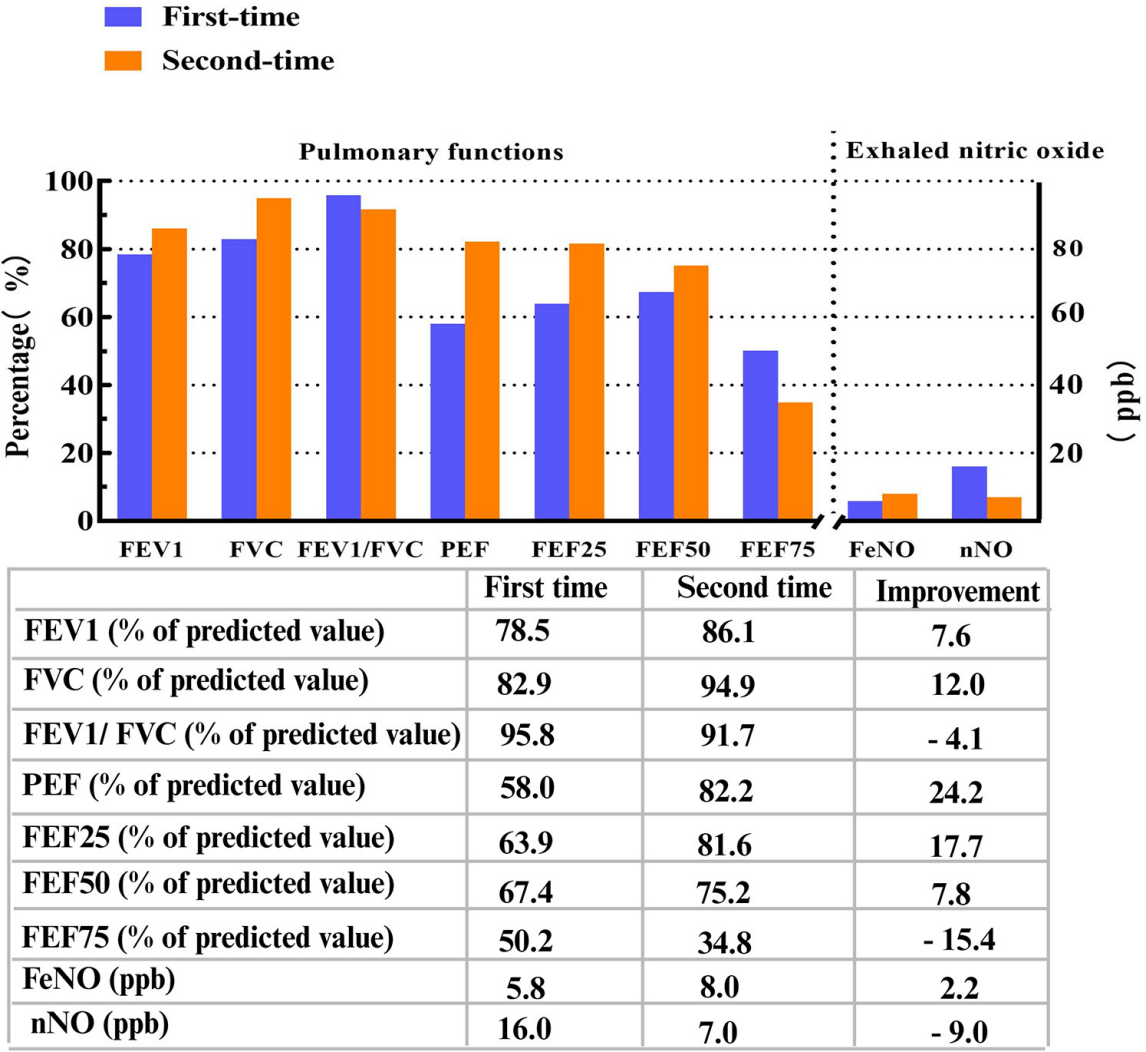
Pulmonary functions and fractional nitric oxide examination detections. FEV_1_, FVC, FEV_1_/FVC, PEF, FEF25, FEF50, and FEF75 were all identified as percentages of predicted values. FEV_1_/FVC ratio, percent of predicted value of FEV_1_ to FVC ratio; FEV_1_, forced expiratory volume in 1 s; FVC, forced vital capacity; PEF, peak expiratory flow rate; FEF25, forced expiratory flow at 25% of forced vital capacity; FEF50, forced expiratory flow at 50% of forced vital capacity; FEF75, forced expiratory flow at 75% of forced vital capacity; FeNO, fractional exhaled nitric oxide; FnNO, fractional nasal nitric oxide. FeNO and FnNO were indicated as ppb.

A lung CT scan indicated infections in the middle lobe of the right lung and the lower lingual segment of the upper lobe of the left lung and inflammation of the lower lobes of both lungs, with slightly dilated bronchi in the lower lobes of both lungs (Figures 1D–F). The first-time pulmonary function (PF) test showed increased airway resistance and decreased ventilation function of small airways, with forced vital capacity (FVC) at 82.9% of the predicted value, forced expiratory capacity in 1 s (FEV_1_) at 78.5% of the predicted value, peak expiratory flow rate (PEF) at 58.0% of the predicted value, and forced expiratory flow at 75% of forced vital capacity with 50.2% of the predicted value. Multiplex combined detection of respiratory pathogens testing of BALF was positive for *P. aeruginosa* DNA and *Streptococcus pneumoniae* DNA and negative for *Mycobacterium tuberculosis* DNA. Her fractional exhaled nitric oxide (FeNO) level was 5.8 ppb. The nasal nitric oxide (nNO) was measured at 16.0 ppb (9.6 nl/min). The patient no longer had a fever following 1 week of intravenous ceftriaxone infusion, 3 days of oral azithromycin anti-infection treatment, and 5 days of glucocorticoid inhalation; however, the patient continued to experience recurrent wet coughing. The second-time PF test exhibited that the flow velocity of medium and small airways decreased, and the function of small airways was impaired. Re-examination showed FVC at 94.9% of the predicted value, FEV_1_ at 86.1% of the predicted value, PEF at 82.2% of the predicted value, and forced expiratory flow at 75% of forced vital capacity with 34.8% of the predicted value. The second time of FeNO measurement was 8.0 ppb, and nNO was 7.0 ppb (4.2 nl/min). PFs and fractional nitric oxide examination results are illustrated in Figure 2.

### Diagnosis

Given the previous experience of full-term born, neonatal pneumonia, prolonged nasal obstruction, purulent nasal discharge, secretory otitis media, bronchiectasis, and extremely low nNO results, the suspected diagnosis is PCD. In addition, Primary Ciliary Dyskinesia Rule (PICADAR) was applied to the patient who has a recurrent purulent cough starting in early childhood. It was used to predict the likelihood of having PCD based on seven questions (7). The total score of this adolescent patient was 6, whose detailed scoring items were summarized (Supplementary Table S1). Based on four general clinical features, PCD was evaluated using an additional scoring system created by the American Thoracic Society (ATS) score (8). Two criteria for this patient were positive, and the specificity of PCD was 72% (Supplementary Table S2).

### Follow-up

**Figure 3:**
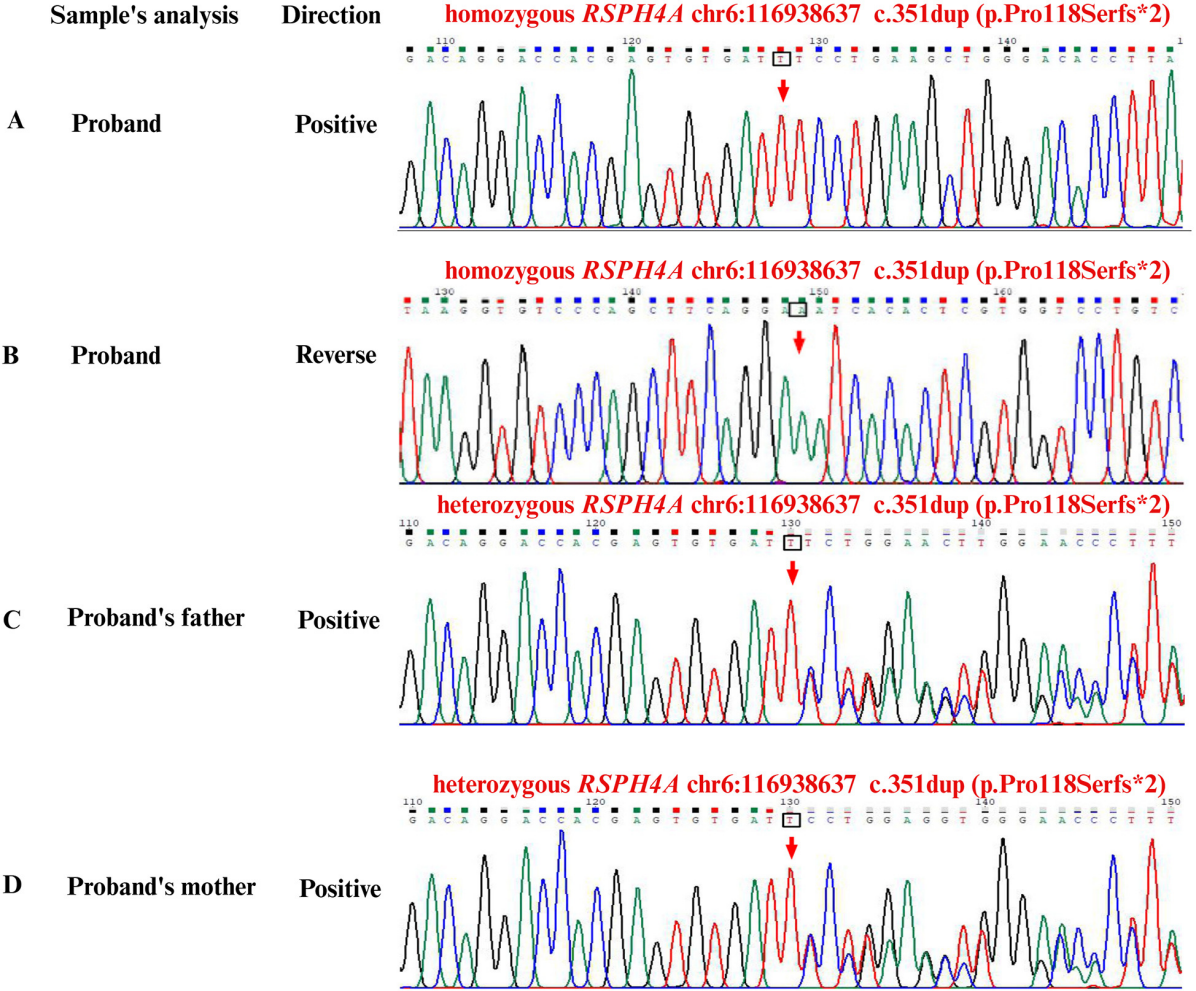
Sanger second-generation sequencing for capturing the whole-exome and gene sequencing. **(A)** Positive sequence of homozygous *RSPH4A*: c.351dup (p.Pro118Serfs*2) frameshift variant in the patient. (B) Reverse sequence of homozygous *RSPH4A*: c.351dup (p.Pro118Serfs*2) frameshift variant in the patient. **(C)** Positive sequence of heterozygous *RSPH4A*: c.351dup (p.Pro118Serfs*2) carrier in the father. **(D)** Positive sequence of heterozygous *RSPH4A*: c.351dup (p.Pro118Serfs*2) carrier in the mother. Green was represented as adenine. Black was represented as guanine. Red was represented as thymine. Blue was represented as cytosine. Hom, homozygous; Het, heterozygous.

Whole-exome sequencing (WES, KingMed Diagnostics, Sichuan, China) verified a homozygous c.351dup (p. Pro118Serfs*2) in the *RSPH4A* gene, approving a definitive diagnosis of PCD (Figure 3). After bronchoscopy, alveolar lavage to clear the respiratory tract, and low-dose oral administration of azithromycin and expectorants, her recurrent wet cough improved. A follow-up examination lung CT scan 2 weeks after discharge revealed multiple bronchiectases with infection in both lungs, which had considerably improved of infections from the initial findings. However, compared with the previous images, there was minimal change in the small patchy ground-glass density shadow in the posterior segment of the right lung's upper lobe. At the 3-month follow-up after discharge, the patient had improved in nasal obstruction, with no recurrent fever or wheezing. Body mass index was appropriate for age and gender. As a measure of inflammation, C-reactive protein was normalized in the blood test to 2.1 mg/L. The patient adhered to the prescribed inhaled corticosteroids and oral montelukast taken daily. No liver function, emotional, or behavioral changes were reported.

## Literature review

Using the keywords “primary ciliary dyskinesia,” “immotile-cilia syndrome,” and “*RSPH4A* gene,” a PubMed literature search was conducted to locate articles published between 2012 and 2025. A total of 12 articles were searched, and clinical characteristics and functional roles of *RSPH4A* mutations in PCD are summarized in Table 1.

**Table 1:** Roles of *RSPH4A* mutations in PCD patients.

Year	Sex	*RSPH4A* variants	Gene mutation roles in PCD	References
2012	Mixed	Q109X, R490X, W356X, IVS3_2–5del	Nonsense and splice site mutations cause central pair absence and microtubular transposition (9 + 0, 8 + 1) in East-Europeans	Zietkiewicz et al. (13)
2013	Mixed	c.921+3_6delAAGT	This mutation is traced back to Hispanic Puerto Ricans and leads to complete loss of function in the respiratory cilia	Daniels et al. (5)
2015	Male	c.166dup (p.Arg56Profs*11)	Novel frameshift mutation causing radial spoke head defects in an Irish Traveler family	Casey et al. (14)
2018	Female	[c.102T>G]+[c.102>G]	NGS-based diagnosis in a Japanese family showing chronic rhinosinusitis and bronchiectasis in PCD	Takeuchi et al. (28)
2020	Mixed	c.430C>T, c.1184A>G, c.1105G>C, and c.2152C>T	WES identified *RSPH4A* mutations in three families in the Turkish cohort; genetic heterogeneity in PCD	Emiralioğlu et al. (15)
2021	Male	c.667delA (homozygous)	Identified in a case with neurofibromatosis type 1; typical PCD symptoms confirmed by TEM	Bian et al. (16)
2021	Mixed	c.921+3_6delAAGT, c.1103T>G	Founder mutation in Puerto Rican descent; causes typical PCD without laterality defects	De Jesús-Rojas et al. (17)
2022	Male	c.2T>C, c.1774_1775del, c.351dupT	Associated with bronchiectasis and infertility; c.2T>C may be a Chinese founder mutation	Wang et al. (18)
2022	Female	c.1105G>C (p.A369P)	Novel missense variant associated with abnormal sperm motility and recurrent infections	Demir Eksi et al. (19)
2022	Male	c.194_224del, c.298delG	Frameshift deletions in *RSPH4A* associated with overlap syndrome of PCD and early respiratory failure	Feng et al. (20)
2024	Female	c.1321T>C (p.Trp441Arg)	Homozygous missense mutation causing chronic cough, otitis media, anosmia, and infertility	Shen et al. (11)
2025	Female	c.1484C>A	Homozygous mutation leading to reduced ciliary beat frequency and central microtubule defects; associated with chronic respiratory symptoms and bronchiectasis	Ogata et al. (24)

## Discussion

PCD is a rare genetic disease characterized by progressive lung disorder, and 54 pathogenic genes have been discovered as causing variants to date (9). In this case, TEM was non-diagnostic, and the final diagnosis was made based on clinical characteristics and WES sequence confirmation, which was in line with diagnostic paradigm for PCD in the *DAW1* gene mutation (10). In a ciliary cross section, nine sets of outer microtubule doublets are usually grouped around a central pair, forming a 9 + 2 ultrastructure. The T-shaped radial spokes join the central pair of microtubules to the outer doublets. *RSPH4A* mutant is an essential structure, as it serves as the central part of the radial spoke head (11). *RSPH4A* variants primarily damage the ciliary ultrastructure and result in the loss of radial spoke heads, which impairs mucus clearance (6).

Previous research revealed that *RSPH4A* mutations are rare in East Asia, accounting for 1.5% of Chinese PCD patients (12). Zietkiewicz et al. (13) firstly reported *RSPH4A* mutations in/around exons 1 and 3, such as *Q109X*, *R490X*, *W356X*, and *IVS3_2–5del*, demonstrating that microtubule transposition phenotype was present in nearly half of the ciliary cross sections examined in patients with *RSPH4A* mutations. Daniels et al. (5) described a novel splice site mutation (c.921+3_6delAAGT) in *RSPH4A*, which could be traced back to Hispanic Puerto Ricans and led to complete loss of function in the respiratory cilia. Casey et al. (14) identified a new mutation in *RSPH4A* (c.166dup; p.Arg 56Pro*11) gene, which caused the loss of all annotated domains, including the radial spoke domain, by introducing an early stop codon at residue 66. Takeuchi et al. conducted a targeted next-generation sequencing panel and discovered a novel disease-causing mutation in exon 1 of *RSPH4A*: [c.102T>G]+[c.102T>G] in nucleotide change. WES identified *RSPH4A* mutations, such as c.430C>T, c.1184A>G, c.1105G>C, and c.2152C>T in three families in the Turkish cohort, and proved genetic heterogeneity in PCD by Emiralioğlu et al. (15). A homozygous mutation c.667delA, p.S223Afs*15 in the *RSPH4A* gene was identified in a case with neurofibromatosis type 1, and typical PCD symptoms were confirmed by TEM (16). De Jesús-Rojas et al. (17) demonstrated a likely pathogenic variant c.1103T>G (p.Val368Gly) in the *RSPH4A* gene, which caused typical PCD without laterality defects. Wang et al. (18) identified heterozygous variants c.2T>C, p.(Met1Thr) and c.1774_1775del, p.(Leu592Aspfs*5) in the *RSPH4A* gene, which were associated with bronchiectasis and infertility, and c.2T>C, p.(Met1Thr) may be a hotspot variant in Chinese. Another novel missense variant c.1105G>C (p.A369P) in the *RSPH4A* gene was also associated with abnormal sperm motility and recurrent infections in PCD, and the t-NGS panel was helpful for efficient and exact identification (19). As for deletion frameshift mutations, Feng et al. (20) revealed c.194_224del and c.298delG in the *RSPH4A* gene, which were also associated with PCD overlap syndrome, as well as early respiratory failure. A novel homozygous mutation c.1321T>C (p.Trp441Arg) in exon 3 of *RSPH4A* was reported by Shen et al. (11), causing symptoms with chronic cough, otitis media, anosmia, and infertility. Prior research indicated that distinct *RSPH4A* mutations consistently led to PCD with specific phenotypic patterns, which were characterized by respiratory symptoms without laterality defects. De Jesús-Rojas et al. discovered that *RSPH4A* (c.921+3_6delAAGT) mutation, as the Puerto Rican founder mutation, demonstrated a very consistent clinical characteristic in several patients, confirming the absence of laterality defects and the presence of typical bronchiectasis and measurable olfactory impairment (21, 22). As Cryoelectron tomography shown by Yoke et al., *RSPH4A KO* mice exhibited a complete absence of all three radial spoke heads (RS1, RS2, RS3) in tracheal cilia. They verified that *RSPH4A* assisted in generating the planar beating of motile cilia by constructing the distal architecture of radial spokes in the trachea, as well as the ependymal tissues (23). The latest homozygous variant c.1484C>A in the *RSPH4A* gene was discovered in Japan, resulting in a reduction in ciliary beat frequency and central microtubule defects, which was also associated with chronic respiratory symptoms and bronchiectasis (24). These indicated that different *RSPH4A* genes led to similar basic clinical symptoms, mainly affecting respiratory epithelia.

**Figure 4:**
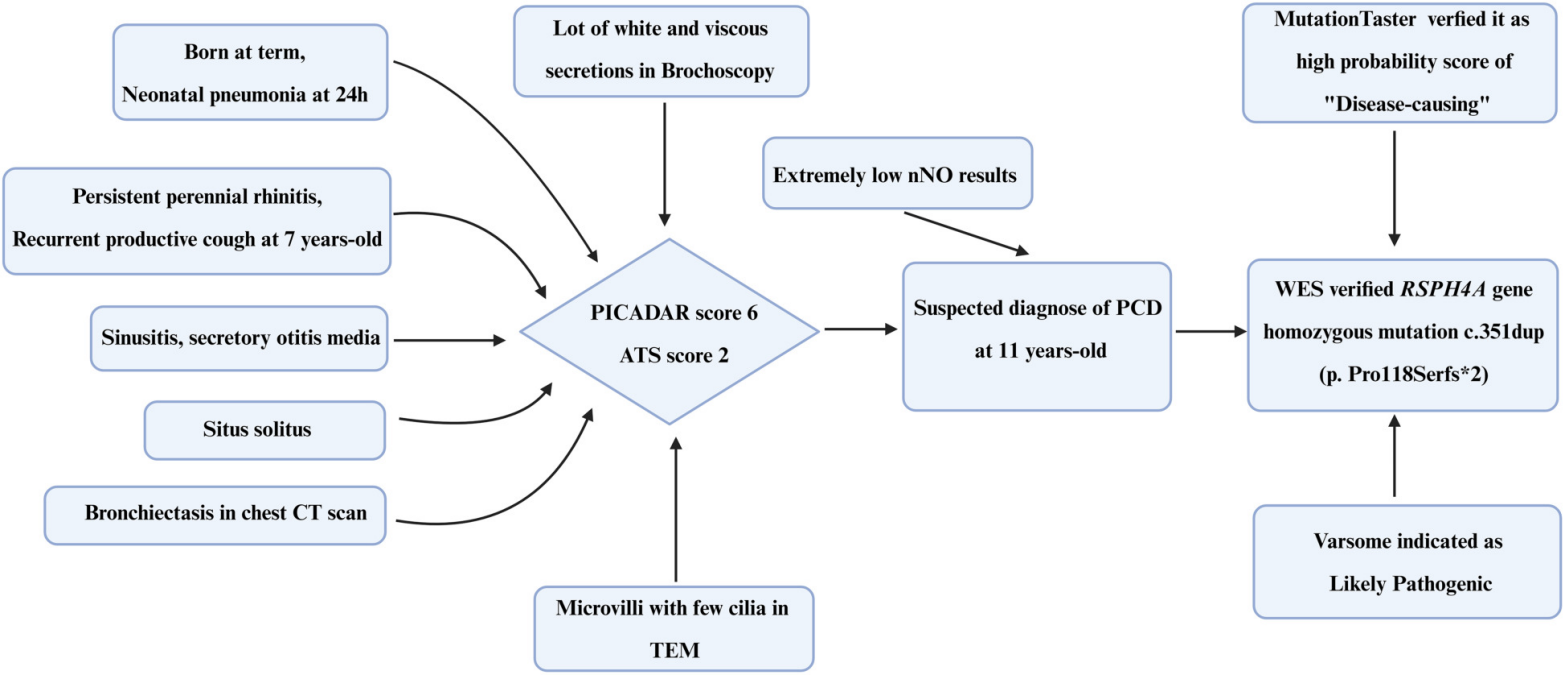
Timeline of the diagnostic process of the PCD patient. PICADAR, Primary Ciliary Dyskinesia Rule; ATS, American Thoracic Society; TEM, transmission electron microscopy; WES, whole-exome sequencing.

In the present research, we analyzed the genetic sequence of an adolescent PCD patient combined with recurrent productive cough, purulent nasal discharge, secretory otitis media, bronchiectasis, and extremely low nNO results. WES results indicated a novel frameshift mutation c.351dup (p. Pro118Serfs*2) in the *RSPH4A* gene. This mutation carried by the proband was inherited from her parents, who carried a heterozygous mutant gene respectively and generated homozygosity in the patient. This frameshift variant c.351dup (p. Pro118Serfs*2) in *RSPH4A* discovered in this adolescent patient has not been reported in the databases or literature yet, including ClinVar (https://www.ncbi.nlm.nih.gov/clinvar), LOVD3 (https://databases.lovd.nl/shared/genes/RSPH4A), and gnomAD (https://gnomad.broadinstitute.org/). All information was searched on 16 May 2025. It is suggested that c.351dup (p. Pro118Serfs*2) in *RSPH4A* is a rare, novel variant, which is first reported in a PCD patient. Additionally, in silico analysis verified that the variant also has an influence on the protein's function. MutationTaster (https://www.genecascade.org/MutationTaster2021/) analyzed the c.351dup (p. Pro118Serfs*2) in *RSPH4A* and identified it as “disease causing” with a high probability score of 1.0. It was suggested that the pathogenic frameshift mutation was pathogenic and could potentially result in nonsense-mediated mRNA decay (NMD). VarSome (https://varsome.com) classified the variant as met PVS1, PM2, and likely PP3, indicating it as likely pathogenic (Supplementary Files 3, 4). Additionally, the timeline of the diagnostic process of the PCD patient is summarized in Figure 4. According to the Genome Aggregation Database (gnomAD, v2.1.1), the *RSPH4A* c.351dup (p.Pro118Serfs*2) variant has not been identified in individuals from diverse global populations, indicating it is a novel frameshift variant with no reported cases to date.

As for pathogens and lung functions in PCD patients, Rubbo et al. (25) indicated that *P. aeruginosa* was isolated from airway samples in adults and induced obviously lower FEV_1_, FVC, and FEF_25%–75%_ *z*-scores, while the results were not very prominent in children. Marthin et al. (26) reported an international consensus on prevention and administration of PCD patients infected with *P. aeruginosa*, which recommended eradication even in asymptomatic situations. Impulse Oscillometry (IOS) was used to assess preschoolers with PCD, and a notable variation in airway resistance and reactance was reported, compared with healthy children (27). In our research, bacterial culture of BALF from this PCD patient exhibited *P. aeruginosa.* She received 1 week of intravenous ceftriaxone infusion and 3 days of oral azithromycin administration so as to eradicate this bacterial infection. Two weeks after discharge, a follow-up lung CT scan revealed that the bilateral lung infections had considerably improved, but multiple bronchiectases still existed.

Apart from these, several limitations in this case deserve to be taken into account. For example, high-speed video microscopy and immunofluorescence staining are recommended in analyzing ciliary ultrastructure for the next step. Combined with the second-generation sequencing results, ciliary functions caused by disease-inducing gene mutation were further identified. Furthermore, long-term monitoring of lung function, exercise evaluation, hearing evaluation, rehabilitation training, and psychotherapy feedback are all necessary to enhance the quality of life for PCD patients.

## Conclusion

We report a case of a PCD patient with a novel *RSPH4A* mutation. Although PCD caused by gene mutation of *RSPH4A* has been reported several times in China, the first case of a pathogenic homozygous frameshift mutation c.351dup (p. Pro118Serfs*2) in the *RSPH4A* gene is reported worldwide. Reporting recently discovered gene mutations is essential for PCD research.

## Data Availability

The original contributions presented in the study are included in the article/Supplementary Material, further inquiries can be directed to the corresponding authors.
