# Supplementary material for: Analysis of clinical and genetic features in an adolescent patient with primary ciliary dyskinesia induced by homozygous mutation in the RSPH4A gene: a case report
Source: Front Pediatr. 2025 Aug 7;13:1630730. doi: 10.3389/fped.2025.1630730 (PMC12367482; doi:10.3389/fped.2025.1630730)
Supplement: Supplementary file 1 [file Datasheet1.pdf]

Supplementary Table 1. PICADAR score of this adolescent patient aged 11

**Does the patient have a daily wet cough that started in early childhood?**		**Yes**
**Questions**	**Answers**	**scores**
1. Was the patient born pre-term or full term?	Full-term	2
2. Did the patient experience chest symptoms in the neonatal period (e.g. tachypnoea, cough, pneumonia)?	Yes	2
3. Was the patient admitted to a neonatal unit?	Yes	2
4. Does the patient have a situs abnormality (situs inversus or heterotaxy)?	No	0
5. Does the patient have a congenital heart defect?	No	0
6. Does the patient have persistent perennial rhinitis?	Yes	1
7. Does the patient experience chronic ear or hearing symptoms (e.g. glue ear, serious otitis media, hearing loss, ear perforation)?	Yes	1
	**Total scores**	**6**

PICADAR, Primary Ciliary Dyskinesia Rule.

Supplementary Table 2. General clinical features of this adolescent patient aged 11 from ATS

**Questions**	**Answers**	**Scores**
Unexplained neonatal respiratory distress	No	0
Early onset, year-round wet cough	Yes	1
Early onset, year-round nasal congestion	Yes	1
Laterality defects	No	0
	**Total scores**	**2**

ATS, American Thoracic Society.

Supplementary File 3

MutationTaster

documentation

mutation t@sting

Prediction disease causing

Model: *complex_aae*, prob: 1 (classification due to NMD, real probability is sho

Summary

- **NMD**
- **amino acid sequence changed**
- **frameshift**
- **protein features (might be) affected**

hyperlink

**analysed issue**	**analysis result**
name of alteration	no title
alteration (phys. location)	chr6:116937999_116938000insG show variant in all transcripts IGV
HGNC symbol	RSPH4A
Ensembl transcript ID	ENST00000229554
Genbank transcript ID	NM_001010892
UniProt peptide	Q5TD94
alteration type	insertion
alteration region	CDS
DNA changes	c.213_214insG<br>cDNA.350_351insG<br>g.358_359insG
AA changes	G73Wfs*47
position(s) of altered AA<br>if AA alteration in CDS	73 (frameshift or PTC - further changes downstream)
frameshift	yes
known variant	Variant was neither found in ExAC nor 1000G.<br>Search ExAC.
regulatory features	DNase1, Open Chromatin, DNase1 Hypersensitive Site<br>H2AZ, Histone, Histone 2A variant Z<br>H3K27me3, Histone, Histone 3 Lysine 27 Tri-Methylation<br>H3K4me2, Histone, Histone 3 Lysine 4 Di-Methylation<br>H3K4me3, Histone, Histone 3 Lysine 4 Tri-Methylation<br>Promoter Associated, Regulatory Feature, Promoter like regulatory feature
phyloP / phastCons	see table below

phyloP / phastCons

	PhyloP	PhastCons
(flanking)	0.786	0.007
(flanking)	0.155	0.006

explain score(s) and/or inspect your position(s) in in UCSC Genome Browser

splice sites	no abrogation of potential splice sites
distance from splice site	351
Kozak consensus sequence altered?	N/A

conservation
protein level for non-synonymous changes

species	match	gene	aa	alignment
Human			73	SPQSRAKTPLGG PAGPETSSPAPVSPREPSSSPSPLAPARQDLAA[illegible]
mutated	partly conserved		73	SPQSRAKTPLGW PRG
Ptroglodytes	all conserved	ENSPTRG00000018535	73	SPQSRAKTPLGG PAGPETSSPAPVSPREPSSSPSPLAPARQDLAA[illegible]
Mmulatta	partly conserved	ENSMMUG00000001805	73	SPQSRARTPLGG PAGPETSSPAPLSRREPSSTPSPPAPARQDLAA[illegible]
Fcatus	no homologue			
Mmusculus	partly conserved	ENSMUSG00000039552	72	SHQSRVSLSTGDLTAGPEVSSSPPPPPLQFHSTPLNTETTQDPVAA[illegible]
Ggallus	no alignment	ENSGALG00000013281	n/a	
Trubripes	no alignment	ENSTRUG00000007282	n/a	
Drerio	no alignment	ENSDARG00000067606	n/a	
Dmelanogaster	partly conserved	FBgn0034957	314	PPAHIGPELTPG IYGWEEFPPEELEKMKPKAAPVPLVEEMELYDI[illegible]
Celegans	no homologue			
Xtropicalis	no alignment	ENSXETG00000019096	n/a	

protein features	start (aa)	end (aa)	feature	details
	370	405	COMPBIAS	Glu-rich. lost
	507	586	COMPBIAS	Glu-rich. lost
length of protein	NMD			
AA sequence altered	yes			
position of stopcodon in wt / mu CDS	2151 / 357			
position (AA) of stopcodon in wt / mu AA sequence	717 / 119			
position of stopcodon in wt / mu cDNA	2288 / 494			
poly(A) signal	N/A			
conservation nucleotide level for all changes - no scoring up to now	N/A			
position of start ATG in wt / mu cDNA	138 / 138			
chromosome	6			
strand	1			
last intron/exon boundary	2054			
theoretical NMD boundary in CDS	1866			
length of CDS	2151			
coding sequence (CDS) position	213 / 214			
cDNA position (for ins/del: last normal base / first normal base)	350 / 351			
gDNA position (for ins/del: last normal base / first normal base)	358 / 359			
chromosomal position (for ins/del: last normal base / first normal base)	116937999 / 116938000			
original gDNA sequence snippet	CTAGAGCCAAGACGCCTCTGGGTGGCCCCGCGGGACCAGA			
altered gDNA sequence snippet	CTAGAGCCAAGACGCCTCTGGGGTGGCCCCGCGGGACCAGA			
original cDNA sequence snippet	CTAGAGCCAAGACGCCTCTGGGTGGCCCCGCGGGACCAGA			
altered cDNA sequence snippet	CTAGAGCCAAGACGCCTCTGGGGTGGCCCCGCGGGACCAGA			
wildtype AA sequence	MEDSTSPKQE KENQEELGET RRPWEGKTAA SPQYSEPESS EPLEAKQGPE TGRQSRSSRP WSPQSRAKTP LGGPAGPETS SPAPVSPREP SSSPSPLAPA RQDLAAPPQS DRTTSVIPEA GTPYPDPLEQ SSDKRESTPH HTSQSEGNTF QQSQQPKPHL CGRRDVSYNN AKQKELRFDV FQEEDSNSDY DLQQPAPGGS EVAPSMLEIT IQNAKAYLLK TSSNSGFNLY DHLSNMLTKI LNERPENAVD IFENISQDVK MAHFSKKFDA LQNENELLPT YEIAEKQKAL FLQGHLEGVD QELEDEIAEN ALPNVMESAF YFEQAGVGLG TDETYRIFLA LKQLTDTHPI QRCRFWGKIL GLEMNYIVAE VEFREGEDEE EVEEEDVAEE RDNGESEAHE DEEDELPKSF YKAPQAIPKE ESRTGANKYV YFVCNEPGRP WVKLPPVIPA QIVIARKIKK FFTGRLDAPI ISYPPFPGNE SNYLRAQIAR ISAGTHVSPL GFYQFGEEEG EEEEEAEGGR NSFEENPDFE GIQVIDLVES LSNWVHHVQH ILSQGRCNWF NSIQKNEEEE EEEDEEKDDS DYIEQEVGLP LLTPISEDLE IQNIPPWTTR LSSNLIPQYA IAVLQSNLWP GAYAFSNGKK FENFYIGWGH KYSPDNYTPP VPPPVYQEYP SGPEITEMDD PSVEEEQAFR AAQEAVLLAA ENEESEEDED EEDDYD*			
mutated AA sequence	MEDSTSPKQE KENQEELGET RRPWEGKTAA SPQYSEPESS EPLEAKQGPE TGRQSRSSRP WSPQSRAKTP LGWPRGTRNI ITCSCLSAGA LFLSFSPGSG QTRPRGTTSV GQDHECDS*			
speed	1.43 s			

Report bugs / help us to improve MutationTaster!

All positions are in basepairs (bp) if not explicitly stated differently.
AA/aa: amino acid; CDS: coding sequence; mu: mutated; NMD: nonsense-mediated mRNA decay; nt: nucleotide; wt: wildtype; TGP: 1000 Genomes Project

Supplementary File 4

chr6-116616973--T (RSPH4A:p.P118Sfs*2)

General Information In... RSPH4A(NM_0010108... p.(Pro118SerfsTer2)	PharmGKB PREMIUM Only available in Premiu...	Germline Classification	Frequencies exomes: ƒ = 0.0000006... genomes: not found (co...	Conservation Scores phyloP100: 0.745	Structural Variants
Genes RSPH4A	Transcripts NM_001010892.3 - fra... MANE Select	ClinVar No data available	MitoMap No data available	In-Silico Predictors No data available	Beacon Network
Community Contributio...	Region Browser	LOVD PREMIUM Only available in Premiu...	Deafness Variation Dat... New No data available	ClinGen No data available	Protein Viewer
Publications Variant: 0 Genes: 23	Expression Data Top: pituitary Tissues: 53	Uniprot Variants No data available	OMIM ® PREMIUM Only available in Premiu...	GWAS No data available	

Variant

Chromosome	Position	REF Sequence	ALT Sequence	Variant type	Cytoband	HGVS
chr6	116616973		T	Insertion	6q22.1	RSPH4A(NM_001010892.3):c.351dup p.(Pro118SerfsTer2)

UCSC genome browser

Mastermind

Gene symbol
RSPH4A

This variant has been viewed **14** times on VarSome.

Connect with past and future viewers of this variant...

Equivalent insertions (2)

chr6-116616974--T TGTGATTTCCTGA
chr6-116616975--T TGTGATTTCCTGA

VarSome.com is for research use only. Find out about our clinically certified platform: VarSome Clinical .

Cite VarSome

Latest News

See all blog posts

Tue, 13 May 2025 12:26:58 GMT
Benchmarking Variant Calling Tools - Wong et al., 2025

Mon, 07 Apr 2025 11:08:23 GMT
World Health Day 2025 - Genomics in Maternal & Newborn Health

Mon, 09 Sep 2024 12:18:58 GMT
Saphetor and Platomics announce agreement to support labs with IVDR compliance

Cite VarSome

VARSOME SUITE

VarSome
VarSome Premium
VarSome Clinical
VarSome API

RESOURCES

Query Examples
Documentation
Germline Classification
Somatic Classification
Global Community
VarSome's Big Data
Increasing Diagnostic Yield

THE COMPANY

About Saphetor SA
News
Cite VarSome!
Contact

SYSTEM STATUS

System status

LEGAL

Terms
Privacy
Information Security

Cookies Settings

VarSome Suite is brought to you by Saphetor SA.

© 2025 Saphetor SA – All Rights Reserved.

Cite VarSome
